# Research on Anchorage Performance of the Foundation Ring for Wind Turbines

**DOI:** 10.3390/ma17081716

**Published:** 2024-04-09

**Authors:** Junjun Zhang, Hao Huang, Li Zhen, Linyuan Sun, Jiaxiang Yang, Kang Chen, Gaixin Chen

**Affiliations:** 1 State Key Laboratory of Simulation and Regulation of Water Cycle in River Basin, Beijing 100038, China; 13889575343@163.com (J.Z.); zhenli@iwhr.com (L.Z.); 18201329224@163.com (L.S.); 13811339072@163.com (J.Y.); chenkang_iwhr@126.com (K.C.); chengx@iwhr.com (G.C.); 2 China Institute of Water Resources and Hydropower Research, Beijing 100038, China; 3 Key Laboratory of Engineering Materials of Ministry of Water Resources, Beijing 100038, China

**Keywords:** FR–foundation connection, numerical simulation, concrete damaged plasticity model, moment capacity, anchorage characteristics

## Abstract

The foundation ring (FR) is a steel component embedded within the concrete of a wind turbine foundation, playing a pivotal role in connecting the wind turbine tower to the foundation structure. In this paper, the FR–foundation connection is equivalent to the exposed foundation and the shallow foundation by analyzing the anchorage characteristics of the foundation ring. Based on the ABAQUS concrete damaged plasticity model, full-scale modeling of the wind turbine foundation is carried out. The influence of embedment depth, ring radius and base flange width of the foundation ring on moment capacity is simulated. Based on the observed stress distributions under ultimate loads, analytical expressions were proposed to estimate the variation law of anchorage load-bearing capacity in the ultimate load state. Compared with the numerical simulation, the average errors under different influencing factors are 8.2%, 9.6% and 10.8%, respectively. The results indicate that the base flange provided the majority of the moment capacity, though the contribution of the sidewall increased to 25–50% that of the base flange in later stages.

## 1. Introduction

Wind turbines are typically supported by gravity-based, reinforced concrete foundations. The foundation ring (FR) is a steel component cast in the concrete of wind turbine foundations that serves as a critical link between the steel tower and the foundation. The FR–foundation connection has advantages such as high stiffness, relatively easy installation and acceleration of construction [1]. The FR–concrete system represents a unique type of structure [2]. With the ongoing development of the wind power industry and an increase in the expected service life of wind turbines, the associated foundation structures have begun to exhibit damage and failure [3,4,5,6,7]. Consequently, researchers around the world have conducted many computational studies to analyze the performance of these structures.

Bai [5] investigated the effects of various parameters on the fatigue life characteristics of concrete foundations for wind turbines using parametric studies and numerical simulations. Lago [8] studied the influence of loading methods and treatment on wind turbine foundations using small-scale models. Saito [9] examined the cracking mechanism and anchorage properties of the “single-pole tower” foundation using experimental data and numerical simulations. Fujiyama [10] analyzed the stability of wind turbine support structures by defining their vibration characteristics using field-monitoring data. Examining the fatigue characteristics of wind turbine foundations, Stavridou [11] found that the diameter of the tower bottom significantly affected fatigue life. He [6] observed the internal stresses in a wind turbine foundation and found that the primary external load was borne by the base flange of the foundation ring. A study conducted by Guo [12] employed a multipoint composite loading system to investigate the actual stress state of foundation structures through simulations using the regional counterweight and elastic foundation methods; the results indicated that the stiffness of the surrounding soil played a critical role in determining the force distribution characteristics of the foundation structure.

Kang [13] and Kang [14] analyzed the mechanical properties of a damaged wind turbine foundation using finite element analyses, finding that the development of a gap between the foundation ring and the surrounding concrete significantly increased the local stress in the concrete. Sano and Ishihara [15] stated that wind turbine foundations primarily fail in the bending moment and conducted a detailed analysis of the factors that influence such failures, including the strength of the concrete and the anchor bolt embedment depth. Zhou [16] found that existing wind turbine foundation designs are susceptible to fatigue damage in the concrete near the base flange of the foundation ring owing to the lack of fatigue strength analyses in that area. Xu [17] simulated the stress–strain state in the foundation ring as well as the anchor cage ring in the surrounding concrete using finite element analyses and proposed a foundation-strengthening scheme based on the results.

In service, the FR–foundation connection bears the full load of the wind turbine superstructure. The FR–concrete system is essential to ensure the safe operation of wind turbine towers. Therefore, a full understanding of the influence of the load-resisting components on the ultimate load-bearing capacity and stress distributions of the FR–foundation connection is urgently needed. At the same time, conducting relevant laboratory tests is challenging due to the considerable size of the wind turbine foundation. Numerical simulations using the finite element method present a reliable alternative to investigating the behavior of the FR–foundation connection of wind turbines under ultimate loading conditions [5,18]. However, existing research on wind turbine foundations has primarily concentrated on fatigue characteristics and alterations in the cracking stress state. Consequently, test data on the load-bearing behavior of the FR–foundation connection are lacking.

In this paper, the FR–foundation connection is equivalent to the exposed foundation and the shallow foundation by analyzing the anchorage characteristics of the foundation ring. The formula of anchorage load-bearing capacity is proposed, and the variation law of anchorage load-bearing capacity under the ultimate load state is analyzed. Taking practical engineering as an example, the concrete damaged plasticity model is adopted to describe the nonlinear behavior of concrete. Three-dimensional (3D) nonlinear numerical models of concrete–foundation ring–steel bar are developed. Detailed parametric studies on the 3D model are carried out to investigate the influence of key parameters (embedment depth, ring radius, flange widths) on the moment capacity and damage of the wind turbine foundation. The accuracy of the proposed theoretical equations is verified by comparing the moment predicted by the theoretical equations with that of the numerical results.

## 2. Calculating the Moment Capacity of the Foundation Ring Structure

### 2.1. Mechanism for Fixing Foundation Rings

**Figure 1:**
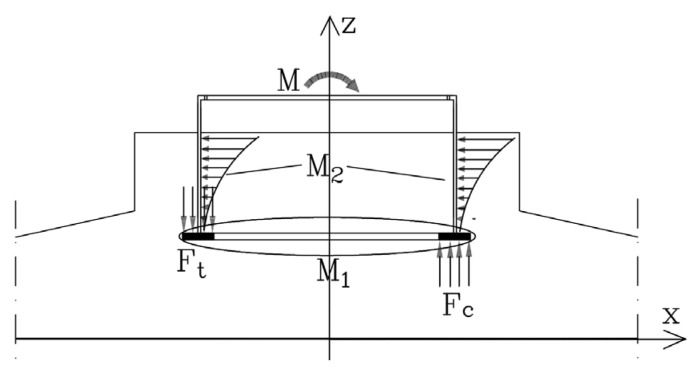
Components of foundation ring moment resistance M: equivalent exposed foundation moment (M_1_) and equivalent shallow foundation moment (M_2_).

The foundation ring is widely utilized in the design of wind turbine foundations due to its exceptional load-bearing capacity and remarkable stability. As shown in Figure 1, two components of foundation ring behavior provide moment capacity: the equivalent exposed foundation moment (M_1_), which is a product of load transfer from the bottom plate of the foundation ring to the concrete above and below; and the equivalent shallow foundation moment (M_2_), which is a product of the shear friction between the foundation ring sidewall and surrounding concrete as well as the comparable horizontal load transfer to the surrounding concrete.

### 2.2. Equivalent Exposed Foundation Moment

**Figure 2:**
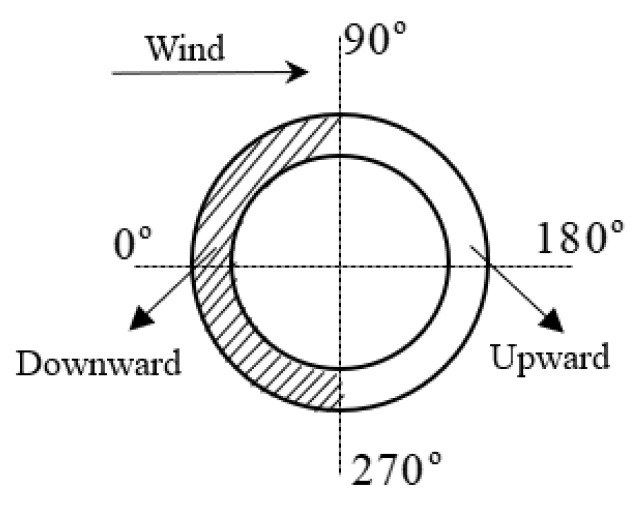
Diagram of the exposed foundation ring forces.

During the operation of a wind turbine, the foundation concrete resists both upward and downward forces to support the foundation ring, as indicated by M1 in Figure 1. As illustrated in Figure 2, the base flange of the foundation ring on the windward side receives a downward reaction force from the concrete, whereas that on the leeward side receives an upward reaction force from the concrete. Thus, opposite sides of the ring base flange experience opposing pressures from the concrete.

**Figure 3:**
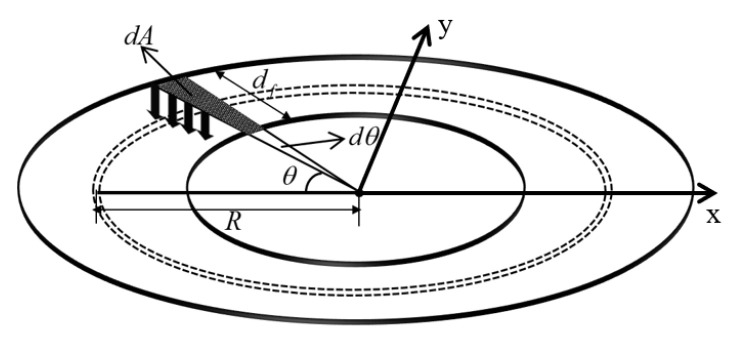
Diagram of the base flange.

According to the relevant study [18], for the base flange at position θ, taking the elemental $dA=R\cdot d\theta \cdot d_{f}\cdot \cos\theta$, the vertical force exerted by the elemental dA is denoted as $dF_{fl}$:

(1) $$dF_{fl}=dA\cdot \sigma_{fl}\cos\theta =Rd_{f}\sigma_{fl}\cos^{2}\theta d\theta ,$$

where $\sigma_{fl}$ is the maximum concrete equivalent force at the middle of the ring base flange width, decreasing from 0° to 90° approximately as a cosine function; $d_{f}$ is the actual width of the ring base flange; and R is the middle radius of the ring base flange (as shown in Figure 3).

**Figure 4:**
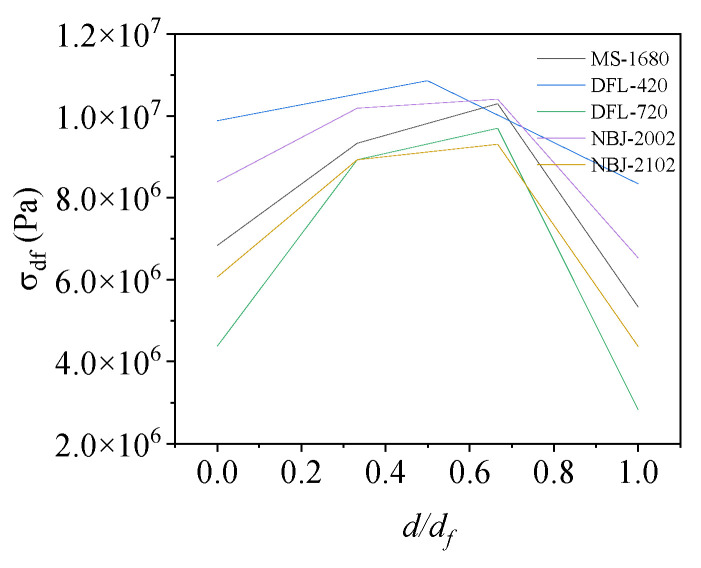
Contact stress curve along the width of the base flange.

Figure 4 illustrates the variation in contact stresses across the width of the base flange. The stress σ(x) of the base flange is not uniformly distributed along the width direction. According to the “area equivalence” principle [19], the effective width of the base flange, $d_{fee}$, is as follows:

(2) $$d_{f}=d_{fee}=\frac{1}{\sigma_{fl}}\int_{0}^{d_{f}}\sigma (x)dx,$$

Thus, the moment capacity of the concrete above and below the ring base flange when subjected to a load can be expressed as follows:

(3) $$M_{\mathrm{dfl}}=4\int_{0}^{\frac{\pi }{2}}R\cos\theta \cdot dF_{fl}=\frac{8}{3}R^{2}\int_{0}^{d_{f}}\sigma (x)dx,$$

### 2.3. Equivalent Shallow Foundation Moment

Under the limit condition, we assume the base flange of the foundation ring can be neglected such that the entire moment is supported by the concrete side walls surrounding the ring. Thus, the foundation ring is only restrained by the pedestal concrete under load, and the system can be simplified as a shallow buried foundation without a base flange, as depicted in the sketch for M_2_ in Figure 1.

**Figure 5:**
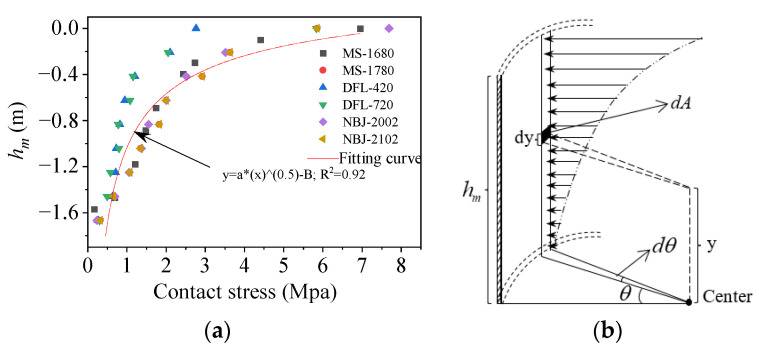
(**a**) Contact stresses at the foundation ring sidewall; (**b**) diagram of the foundation ring sidewall.

Based on the outcomes of the numerical simulation, it is presumed that the sidewall stresses are distributed as a power function along the height direction, as shown in Figure 5. For the microelement dA=R⋅dθ⋅dy, the radial stresses at the θ position on the foundation ring sidewall can be expressed as $\sigma_{\theta }=\sigma_{\max}\cos\theta$, where $\theta \in (0,\frac{\pi }{2})$, and the stress at any height *y* is given by $\sigma_{y}=\frac{{h_{m}}^{2}}{{\left(h_{m}+y\right)}^{2}}\cdot (\sigma_{\max}\cos\theta )$. The following equation accordingly expresses the sidewall bending moment:

(4) Mcb=4∫0hm∫0π2σyydA(cosθ)=15πσmaxRhm2,

where $\sigma_{\max}$ denotes the maximum sidewall stress at 0 degrees on the windward side, decreasing from $0^{o}$ to $90^{o}$ approximately as a cosine function; $h_{m}$ denotes the embedment depth of the foundation ring.

## 3. Numerical Simulations

### 3.1. Finite Element Model

**Figure 6:**
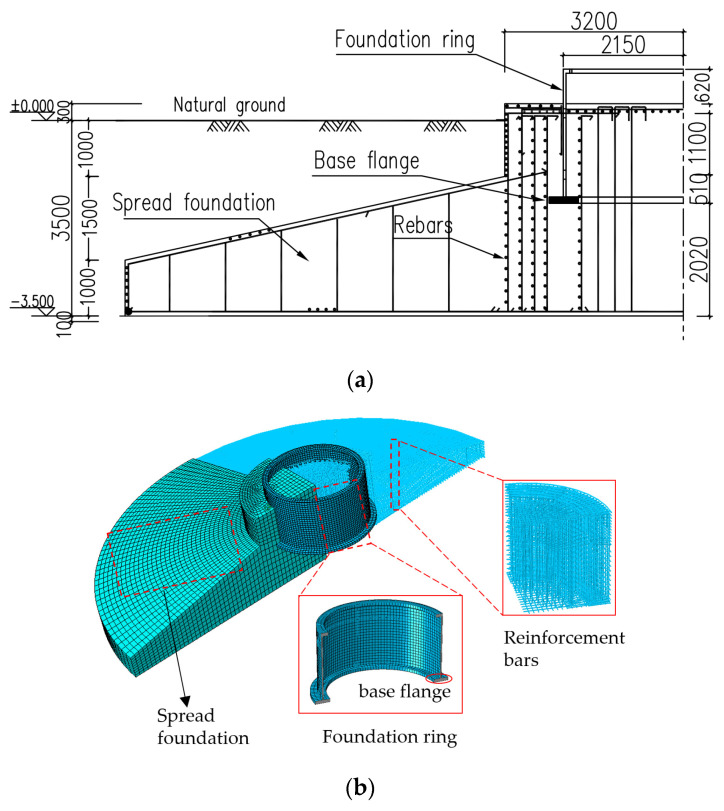
Wind turbine foundation model: (**a**) drawing of cross section; (**b**) finite element model.

The impacts of the foundation ring embedment depth, base flange width, and radius on the capacity, working conditions, and stress characteristics of a wind turbine foundation were investigated using a 2.3 MW wind turbine from an East China wind farm as a case study. Figure 6a shows the case study foundation section and Table 1 presents the considered load conditions (the Z axis is the height direction of the tower).

**Figure 7:**
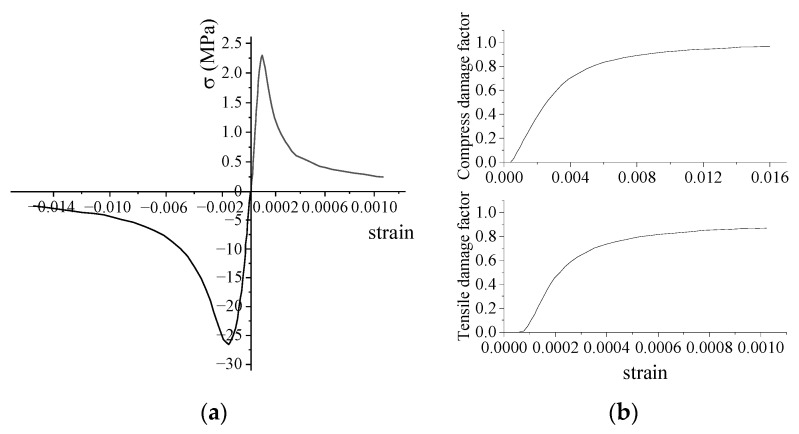
Concrete plastic damage model: (**a**) stress–strain curve; (**b**) damage curve.

In order to investigate the load-bearing capacity behavior of the FR–foundation connection of wind turbines, three-dimensional (3D) nonlinear numerical models of the wind turbine foundation were constructed using ABAQUS 2022 finite element software. The 3D model consists of the foundation, steel reinforcement bars, and the steel ring. The diameters of the circular spread foundation and the foundation ring are 20 m and 4.3 m, respectively. The C3D8R concrete element and T3D2 steel element were used in this model, which is shown in Figure 6b. To simulate the nonlinear behavior of concrete during the stressing process, the stress–strain and damage curves shown in Figure 7 [20,21,22] were utilized along with the concrete damage plasticity model. The coefficient of friction between the steel foundation ring and surrounding concrete was assumed to be 0.55 [23,24]. The foundation ring opening was similar in size to that specified for perforated reinforcement, and not in contact with the concrete reinforcement; the interaction between the concrete, reinforcement, and foundation ring was simulated using the “embedded region” command. The moment was applied to the top surface of the foundation ring at the center of the circular coupling point. The material parameters applied in the model are listed in Table 2.

### 3.2. Parametric Analysis and Verification of Equations Using Simulation Results

A parametric analysis of the foundation ring moment capacity was conducted by varying the dimensional parameters in a series of finite element models as described in Table 3 and Table 4. The base flange moment capacity and sidewall moment capacity are denoted by the letters *M*_dfl_ and *M*_sw_, respectively. The moment capacity results were subsequently applied to verify the accuracy of Equations (3) and (4). The results are reported in Table 4. α is the rotation angle of the foundation ring and η describes the average relative error of the calculated ring base flange moment, respectively defined as

(5) $$\alpha =\arcsin\frac{\delta_{t}-\delta_{c}}{D},$$

(6) $$\eta =\frac{1}{n}\sum_{i=1}^{n}\frac{M_{1}-M_{2}}{M_{2}},$$

where $\delta_{t}$ and $\delta_{c}$ represent the vertical displacements of the ring base flange at 0° and 180° (on the windward and leeward sides), respectively; *D* is the outer diameter of the ring base flange; $M_{1}$ is the calculated moment capacity; $M_{2}$ is the simulated moment capacity (considered to be the “true” value in this study).

**Figure 8:**
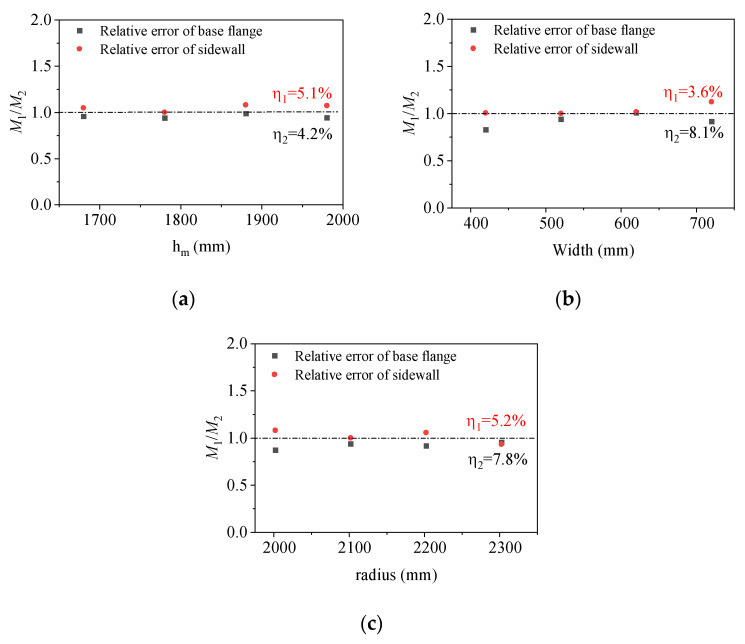
Relative errors of different parameter models: (**a**) embedment depth ratio; (**b**) base flange width; (**c**) foundation ring radius.

The scatter plots of the relative errors for the ultimate moment capacities at different burial depth ratios, base flange widths, and foundation ring radius are shown in Figure 8. The plots indicate that there is little variation in relative errors across different parameter values. The average relative error for the moment capacity is within 10%.

#### 3.2.1. Effect of Foundation Ring Embedment Depth on Moment Capacity

**Figure 9:**
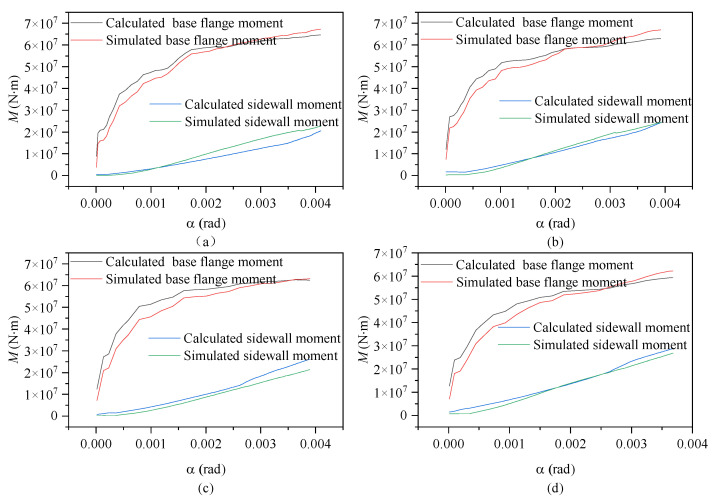
Moment–rotation curves according to foundation ring embedment depth: (**a**) model MS-1680; (**b**) model MS-1780; (**c**) model MS-1880; (**d**) model MS-1980.

Figure 9 shows the calculated and simulated moment–rotation curves for the contributions of the base flange (equivalent exposed foundation moment) and sidewall (equivalent shallow foundation moment) according to embedment depth. Throughout the entire loading process, the moment contributions of each component exhibited a consistent pattern regardless of embedment depth. As the rotation angle increased, both the base flange and sidewall concrete exhibited gradual increases in moment resistance, albeit following different trends, which is consistent with the real-world scenario.

During the initial loading phase, the concrete in the base flange undergoes elastic deformation, leading to a rapid increase in load-bearing capacity. As the load continues to escalate, localized damage becomes apparent in the base flange concrete, resulting in a growing disparity between the two. Accounting for the impact of the effective width of the base flange on load-bearing capacity, numerical simulations accurately depict the effective width of the base ring flange; as damage accumulates, sections of the T-shaped flange cease to contribute to structural calculations.

Theoretical equations assume the involvement of the T-shaped flange in stress distribution, thereby inflating the effective width of the T-shaped flange and yielding conservative calculation results. Although this approach introduces a certain deviation from reality, the error remains within an effective and reasonable range. At the maximum rotation, the sidewall moment capacity reached approximately 33~42% of the base flange moment capacity under the various foundation ring embedment depths. The general agreement of the calculated and simulated results indicates that Equations (3) and (4) presented in this study can accurately reflect the changes in wind turbine foundation moment capacity according to the foundation ring embedment depth.

**Figure 10:**
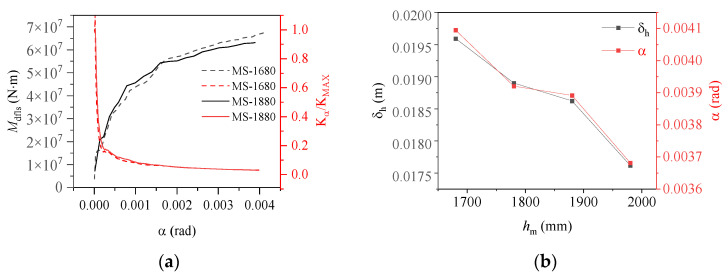
Changes in (**a**) moment capacity and stiffness with rotation according to embedment depth; (**b**) maximum vertical displacement and rotation according to embedment depth.

The stiffness of the base flange, denoted as $K_{\alpha }=M_{\alpha }/\alpha$, was “normalized” to better illustrate the variation in the moment capacity of the base flange concrete under load, as shown in Figure 10a. The stiffness of the ring base flange was observed to change according to the embedment depth following a consistent law: during the early loading stage, the load resistance characteristics resembled those for an equivalent exposed foundation, with the primary load carried by the ring base flange, resulting in a rapid reduction in stiffness; as the load increased, the base flange concrete experienced localized damage, and as the foundation ring continued to rotate, a portion of the base flange load-bearing capacity was transferred to the sidewall concrete. Consequently, the stiffness of the ring base flange began to decrease at a slower rate, and the load resistance characteristics of the foundation structure began to resemble those of an equivalent shallow foundation. As shown in Figure 10b, an increase in the embedment depth of the foundation ring resulted in a gradual increase in the moment capacity of the sidewall concrete as it increasingly restricted the rotation of the foundation. Consequently, the vertical displacement ($\delta_{h}$) gradually decreased and eventually reached an equilibrium state.

#### 3.2.2. Effects of Foundation Ring Radius on Moment Capacity

**Figure 11:**
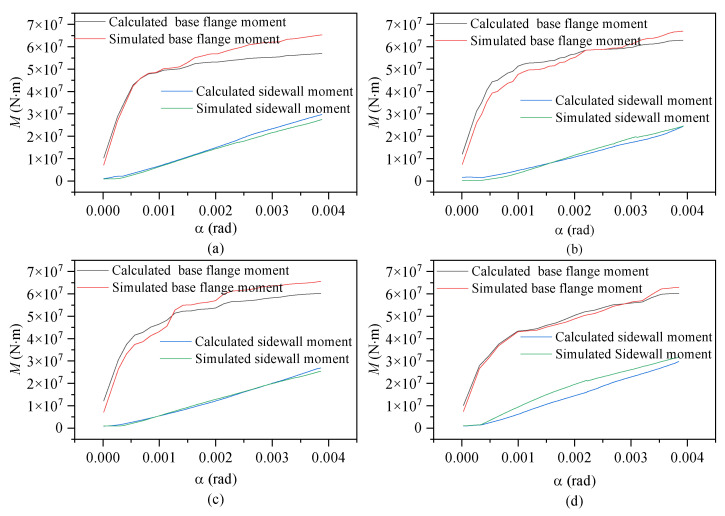
Moment–rotation curves according to foundation ring radius: (**a**) model NBJ-2002; (**b**) model NBJ-2102; (**c**) model NBJ-2202; (**d**) model NBJ-2302.

**Figure 12:**
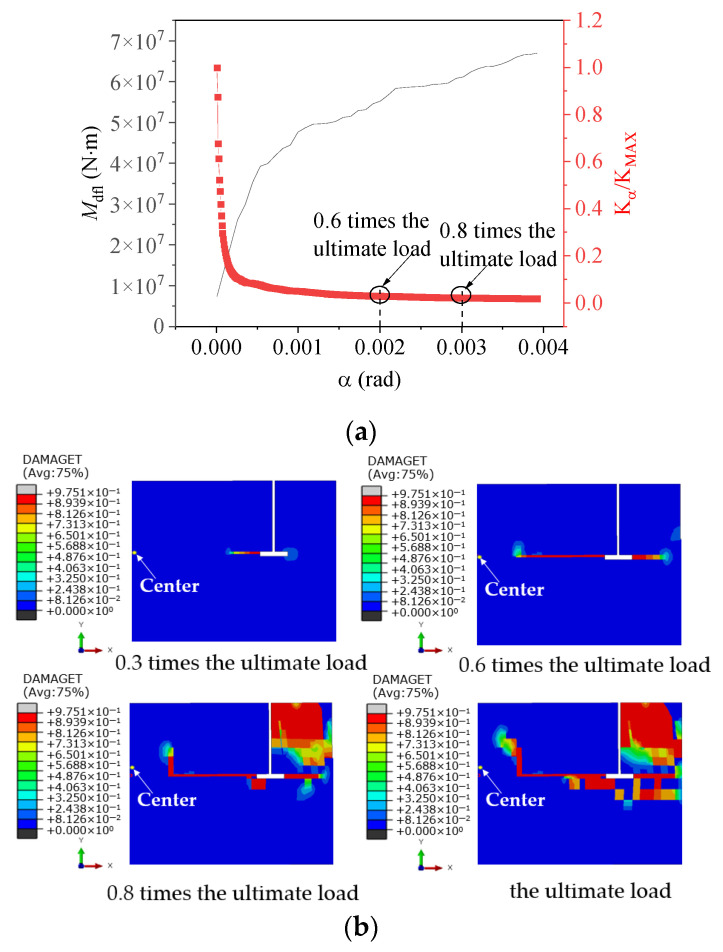
Relationship between damage and base flange moment capacity for specimen NBJ-2102: (**a**) moment–rotation curve; (**b**) cloud diagram of foundation concrete damage.

Figure 11 and Figure 12 show the relationships between the moment capacity and rotation angle of the foundation ring according to the foundation ring radius. The figures indicate that the moment capacity of the base flange increased gradually as the applied load increased. Once the foundation ring rotated to a specific angle, the concrete near the base flange was damaged, causing the rate of increase in the moment capacity of the base flange to slow and the sidewall concrete to resist more of the moment at a faster rate. Overall, the calculation results were in good agreement with the results of the numerical simulations, indicating that the proposed equations for the concrete moment capacity of a wind turbine foundation accurately captured the changes according to the foundation ring radius. At the maximum rotation, the sidewall moment capacity reached approximately 38~50% of the base flange moment capacity under the various foundation ring radius.

Figure 12 illustrates the variation in concrete damage on the windward side of the foundation ring at different load levels, as well as the change in moment capacity with the rotation of the model NBJ-2102 base flange. During the initial loading phase, the majority of the external load was carried by the base flange concrete, leading to a rapid degradation of stiffness. The stiffness of the ring base flange subsequently exhibited relatively minor variations after a rotation of 0.002 rad. As the experiment progressed, cracking damage eventually migrated toward the middle of the foundation ring, while the concrete damage near the base flange worsened, causing the onset of spalling at the bottom of the ring. The sidewall concrete began to play a more significant role once the load reached 0.8 times the ultimate load. At this time, the rotation of the foundation ring began to increase at a faster rate as the damage to the sidewall concrete worsened. The stiffness of the base flange remained nearly flat while the damaged area increased slightly; the bending moment in the base flange concrete exhibited an increase comparable to that in the sidewall concrete. Finally, a significant area of damage emerged near the ring base flange and on the outer concrete of the sidewall.

**Figure 13:**
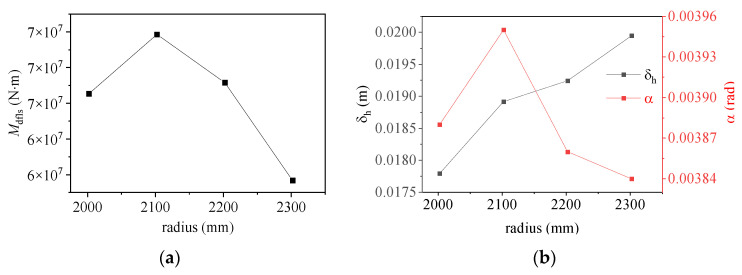
Effect of base flange radius on (**a**) moment capacity; (**b**) vertical deflection and rotation.

Figure 13 shows that the base flange moment capacity does not vary linearly with the radius; rather, it exhibits a certain “size effect”. Based on the results of numerical simulations, it was observed that the moment capacity of the base flange gradually increased when the radius was less than 2100 mm. However, when the radius exceeded 2100 mm, the increase in contact area resulted in the concrete stress predominantly affecting the moment capacity, leading to a gradual decrease in the base flange moment capacity. Because of an increase in the foundation ring radius, the vertical displacement gradually increased, albeit at a diminishing rate. Therefore, all relevant factors must be considered when optimizing specific parameters to accurately determine the appropriate dimensional specifications for a given project.

#### 3.2.3. Effect of Base Flange Width on Moment Capacity

**Figure 14:**
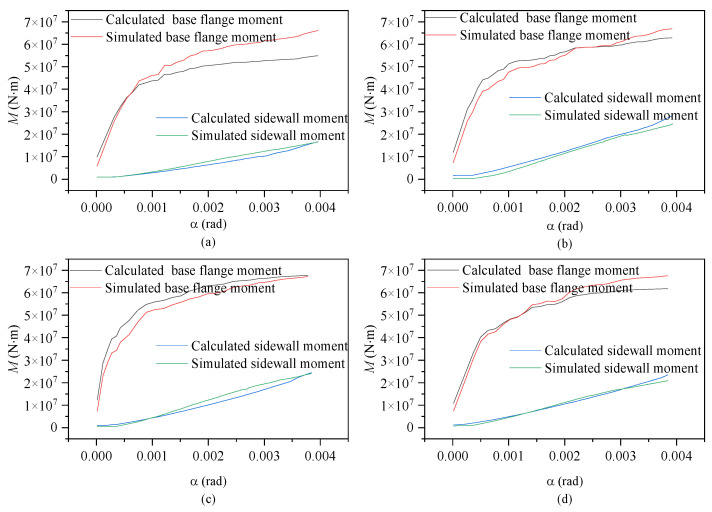
Moment–rotation curves according to base flange width: (**a**) model DFL-420; (**b**) model DFL-520; (**c**) model DFL-620; (**d**) model DFL-720.

**Figure 15:**
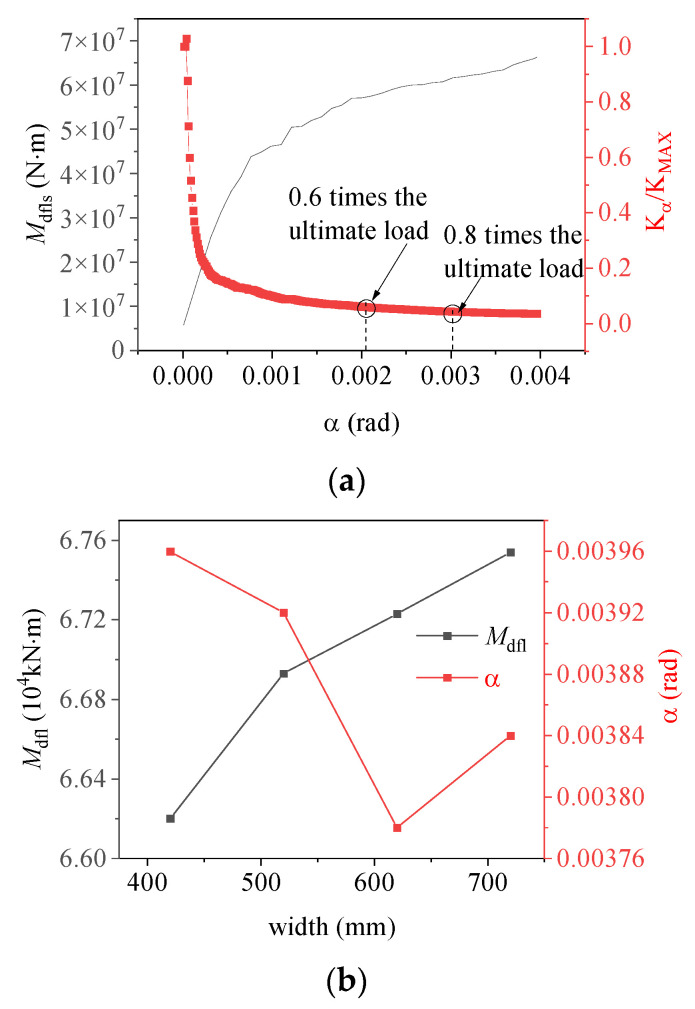
(**a**) Base flange moment capacity and stiffness according to the rotation for model DFL-420; (**b**) moment capacity and rotation according to flange width.

Figure 14 and Figure 15 show the relationships between the moment capacities and rotation angle of the foundation ring according to the base flange width. During the initial loading stage, the moment capacity of the base flange increased rapidly, but slowed upon the “intervention” of the sidewall concrete. At 0.8 times the ultimate load, the stiffness of the base flange concrete reached a steady state. The results of the numerical simulation and calculations using the proposed equations were in general agreement, indicating that the change in moment capacity with rotation remained consistent regardless of the base flange width. At the maximum rotation, the sidewall moment capacity reached approximately 25~36% of the base flange moment capacity under the various base flange widths.

As illustrated in Figure 15b, the base flange moment capacity steadily increased as the flange width increased; however, the rate of increase slowed, indicating diminishing influence. The rotation of the foundation ring increased once the width of the base flange exceeded 620 mm, potentially because local stress concentrations damaged the base flange concrete. Consequently, increasing the width of the base flange alone does not increase the safety of the foundation.

**Figure 16:**
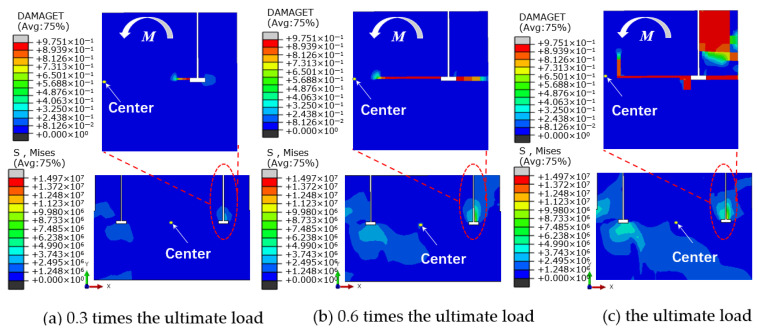
Concrete foundation stress and damage clouds for model DFL-420.

**Figure 17:**
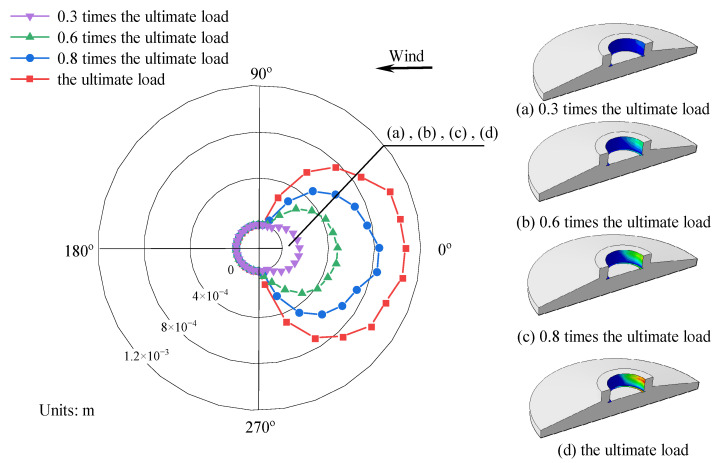
Concrete sidewall separation for model DFL-420.

Figure 16 depicts the stresses and damage in model DFL-420. During the initial loading phase, when the external load was transmitted through the foundation ring, stress concentrations occurred on the compression and tension sides of the base flange concrete, as shown in Figure 16a. As illustrated in Figure 17a,b, the base flange bore most of the external load at this time, and there were visible signs that the concrete sidewall on the windward side began to detach from the foundation ring. As the load increased further, the stresses in the concrete around the base flange spread outward in a “triangular” pattern. Concurrently, the gap between the foundation ring and concrete sidewall gradually widened, starting from the windward (0°) side and decreasing symmetrically in both directions, as shown in Figure 17c,d. Once the damage reached a certain level, localized concrete failure occurred near the base flange, and the crack damage gradually expanded toward the center of the foundation, as shown in Figure 16b. The high-stress zone in the sidewall concrete gradually expanded, and the moment capacity of the base flange gradually decreased until equilibrium was reached, as shown in Figure 16c.

Figure 17 shows the separation between the concrete at the top of the sidewall and the steel foundation ring. An increase in the load can be observed to have gradually caused a symmetrical, fan-shaped expansion of the separated area along the foundation ring sidewall between the −90° and 90° directions. Both the separation depth and distance gradually increased as well, with the latter reaching 1 mm. The concrete on the leeward side remained in contact with the foundation ring throughout loading.

## 4. Conclusions

In this paper, taking practical engineering as an example, detailed parametric studies on a 3D model were carried out to investigate the influence of key parameters (embedment depth, ring radius, flange widths) on the moment capacity and damage of the wind turbine foundation. The following conclusions were drawn from this study.

(1) The concrete damage laws among foundation ring models of varying sizes are largely consistent. Initially, a plastic damage zone is developed within the concrete near the T-shaped flange. As the bending moment increases, the damage zone gradually expands. At 0.3 times the ultimate load, the plastic damage area gradually extends towards the “centroid” of the foundation ring and the surrounding outer concrete. At this time, the stiffness of the concrete near the T-shaped flange starts to diminish, while the load-bearing capacity of the side wall concrete gradually increases. There is a sharp increase in concrete damage along the side wall. At the ultimate load, the damaged area of concrete on the side wall reaches the maximum extent, rendering the foundation susceptible to punching shear failure.

(2) Under different embedment depth conditions, the theoretical equations effectively capture the variation in the foundation’s load-bearing capacity. Compared with the numerical simulation results, the average error is less than 8%. For the foundation ring models with different embedment depths, the moment capacity of the side wall constitutes less than 42% of that of the base flange. As the embedment depth increases, both the rotation angle and vertical displacement of the foundation ring decrease gradually. When the rotation angle exceeds 0.001 radians, the stiffness of the concrete near the base flange remains nearly constant, indicating a gradual stabilization of the structure.

(3) Under different ring radius conditions, the average error between the theoretical equation calculation results and the numerical simulation results remains within 10%. For the foundation ring models with different ring radii, the moment capacity of the side wall constitutes less than 50% of that of the base flange. As the ring radius increases, the angle curve of the foundation ring exhibits a “single peak” state. The vertical displacement increases gradually, with the rate of increase also showing a gradual rise. When the rotation angle exceeds 0.002 rad, the stiffness of the concrete near the base flange remains nearly constant, indicating a gradual stabilization of the structure.

(4) Under different base flange width conditions, the theoretical equations effectively capture the variation in the foundation’s load-bearing capacity. Compared with the numerical simulation results, the average error is less than 10.8%. For the foundation ring models with different embedment depths, the moment capacity of the side wall constitutes less than 42% of that of the base flange. As the width increases, the rotation angle of the foundation ring reaches its minimum value. The concrete on the windward side becomes detached from the foundation ring. With $0^{o}$ as the “central reference point”, it gradually expands in a fan shape and extends downward from the foundation surface.

(5) The moment capacity of the foundation was influenced by the ring radius, flange width, and embedment depth of the base flange. The base flange concrete provided the majority of the moment capacity; the moment capacity of the sidewall represented approximately 25~50% that of the base flange. However, each of these parameters has practical limitations when changed to improve the moment capacity in engineering applications, and the interactions between these parameters and the overall foundation structure must be considered. The actual design of a wind turbine foundation must assume that the base flange will carry all loads to guarantee the safe operation of the structure.

## Figures and Tables

**Table 1:** Load conditions.

Loading Condition	Safety Factor	F_Z_ (kN)	F_x−y_ (kN)	M_Z_ (kN·m)	M_x−y_ (kN·m)
Extreme load	1.0	−3176.1	874	1921	81,811

**Table 2:** Parameters of the concrete foundation components.

Models	Elastic Modulus (GPa)	Poisson Ratio/$v_{c}$	Density (kg·m^−3^)	Compress/Tensile Strength (MPa)	Dilation Angle	Eccentricity	Viscosity Parameter
C40	32.5	0.2	2500	26.8	38	0.1	$10^{-5}$
HRB400	200	0.3	7850	360	-	-	-

**Table 3:** Numerical simulation parameters.

Model ID	Parameter			
	*d_f_* (mm)	*h_m_* (mm)	*R*_n_ (mm)	*h_m_*/*D*
MS-1680	520	1680	2102	0.39
MS-1780	520	1780	2102	0.42
MS-1880	520	1880	2102	0.44
MS-1980	520	1980	2102	0.47
DFL-420	420	1780	2102	0.42
DFL-520	520	1780	2102	0.42
DFL-620	620	1780	2102	0.42
DFL-720	720	1780	2102	0.42
NBJ-2002	520	1780	2002	0.44
NBJ-2102	520	1780	2102	0.42
NBJ-2202	520	1780	2202	0.4
NBJ-2302	520	1780	2302	0.38

“MS” is the foundation ring embedment depth code; “NBJ” is the foundation ring radius code; “DFL” is the base flange width code.

**Table 4:** Numerical simulation results.

Model ID	Simulation Results				Equations Results	
	α (rad)	*δ_h_* (m)	*M*_dfls_ (kN⋅m)	*M*_sws_ (kN⋅m)	*M*_dfls_ (kN⋅m)	*M*_sws_ (kN⋅m)
MS-1680	0.00409	0.0196	67,290	22,646	64,573	23,750
MS-1780	0.00392	0.0189	66,930	24,610	62,909	24,618
MS-1880	0.00389	0.0187	63,170	21,369	62,415	23,101
MS-1980	0.00368	0.0176	63,030	26,811	59,380	28,815
DFL-420	0.00396	0.0185	66,200	16,611	54,967	16,705
DFL-520	0.00392	0.0189	66,930	24,610	62,909	24,618
DFL-620	0.00378	0.0184	67,230	24,090	67,800	24,492
DFL-720	0.00384	0.0191	67,540	20,917	61,817	23,496
NBJ-2002	0.00388	0.0178	65,270	27,476	56,962	29,745
NBJ-2102	0.00392	0.0189	66,930	24,610	62,909	24,618
NBJ-2202	0.00386	0.0192	65,580	25,420	60,220	26,900
NBJ-2302	0.00384	0.0199	62,840	31,782	60,133	29,708

## Data Availability

Data are contained within the article.
